# Severe necrotizing soft-tissue infection-associated mortality: Have a look at the computed tomography!

**DOI:** 10.1186/s13054-022-03898-1

**Published:** 2022-01-25

**Authors:** Sébastien Tanaka, Michael Thy, Ralph Khoury, Alexy Tran-Dinh, Antoine Khalil, Philippe Montravers

**Affiliations:** 1 Assistance Publique - Hôpitaux de Paris (AP-HP), Department of Anesthesiology and Critical Care Medicine, DMU PARABOL, Bichat-Claude Bernard Hospital, 46 Rue Henri Huchard, 75018 Paris, France; 2 French Institute of Health and Medical Research (INSERM), U1188 Diabetes Atherothrombosis Réunion Indian Ocean (DéTROI), CYROI Plateform, Réunion Island University, Saint-Denis de La Réunion, France; 3 Université de Paris, Paris, France; 4 EA 7323 - Pharmacology and Therapeutic Evaluation in Children and Pregnant Women, Paris Descartes University, Sorbonne Paris Cité University, Paris, France; 5 Assistance Publique - Hôpitaux de Paris (AP-HP), Department of Radiology, DMU DREAM, Bichat-Claude Bernard Hospital, Paris, France; 6 Laboratory for Vascular Translational Science, French Institute of Health and Medical Research (INSERM) U1148, Paris, France; 7 PHERE, Physiopathology and Epidemiology of Respiratory Diseases, French Institute of Health and Medical Research (INSERM) U1152, Paris, France

**Keywords:** Necrotizing soft-tissue infection, Sepsis, Outcome, Intensive care unit, Computed tomography

Necrotizing soft-tissue infection (NSTI) is a life-threatening pathology, and the cornerstone treatment is based on early diagnosis, surgical source control and antimicrobial therapy [1]. Even if the diagnosis remains essentially clinical, computed tomography (CT) could be helpful in the diagnosis but remains controversial [2]. To date, there are no data screening the criteria for the place of initial CT-scan and patient outcomes. We aimed to evaluate the relationship between CT signs and the outcome of severe NSTI patients.

We retrospectively collected data for 100 patients with severe NSTI hospitalized in our intensive care unit (ICU) between 2009 and 2019 and whose diagnoses were surgically confirmed. Methods of this cohort have been previously published [3]. Patients who were clinically suspected of having NSTI benefited from urgent surgical exploration. CT was performed prior to surgery at the discretion of the clinician if the diagnosis of NSTI was uncertain or to assess the extent of the damage.

Four criteria for CT were evaluated according to previous guidelines [4, 5]:

- Gas in the soft tissues,
- Multiple fluid collections,
- Absence or heterogeneity of tissue enhancement by IV contrast,
- Significant inflammatory changes of the fascia and under.

The presence of these criteria was compared between survivors and nonsurvivors at day-90.

Of the 100 patients, 54 underwent CT before surgical exploration. Table 1 shows the comparison of general characteristics between the patients with and without CT. The median (IQR) delay between CT and the surgical procedure was 5 [2–20] hours. Table 2 shows the clinical and CT characteristics according to the survival or death status at day-90. In the multivariate analysis, in addition to the surface parameter (OR 1.15, 95% CI 1.34, *p* = 0.01), the criterion for inflammatory changes of the fascia was associated with mortality at day-90 (OR 8.09, 95% CI 63.5, *p* = 0.015). Inflammatory changes of the fascia parameter had a sensitivity of 60% (32-84), specificity of 92% (79-98), positive predictive value of 75% (43–95) and negative predictive value of 86% (71–95).

**Table 1:** Overall baseline and outcome features of the study patients according to CT evaluation

	Overall *n* = 100	No CT *n* = 46	CT *n* = 54	*p* value
Age (years), median [IQR]	58 [50–68]	57 [48–68]	59 [53–68]	0.31
Male sex, *n* (%)	63 (63)	26 (56.5)	37 (68.5)	0.299
BMI (kg/m^2^), median [IQR]	28 [23–34]	29 [26–37]	25 [22–32]	0.059
Localization: limbs, *n* (%)	52 (52)	35 (76)	17 (31)	< 0.001
Localization: pelvis, *n* (%)	28 (28)	12 (26)	16 (30)	0.824
Localization: cephalic, *n* (%)	23 (23)	10 (22)	13 (24)	0.97
Localization: trunk, *n* (%)	13 (13)	1 (2)	12 (22)	0.008
Skin surface (%), median [IQR]	4.5 [4.5–9]	4.5 [4.5–9.0]	4.50 [4.5–9.0]	0.467
SAPS-II on admission, median [IQR]	28 [23–37]	33 [25–44]	39 [23–56]	0.369
SOFA score on admission, median [IQR]	5 [3–6]	4 [1, 7]	4 [1, 9]	0.721
LRINEC score on admission, median [IQR]	2 [1–4]	3 [2, 5]	3 [1, 5]	0.709
Septic shock on admission, *n* (%)	65 (65)	30 (65)	35 (65)	1
Day-1 cardiovascular failure (HD SOFA ≥ 1), *n* (%)	87 (87)	42 (91)	45 (83)	0.372
Day-1 respiratory failure (respiratory SOFA ≥ 1), *n* (%)	44 (44)	20 (44)	24 (44)	1
Day-1 renal failure (renal SOFA ≥ 1), *n* (%)	42 (42)	18 (39)	24 (44)	0.685
Charlson score on admission, median [IQR]	4 [3–7]	3 [2–5]	5 [3–7]	0.008
Underlying medical conditions				
Malignancy, *n* (%)	23 (23)	7 (15)	16 (30)	0.101
Obesity, *n* (%)	24 (24)	13 (28)	11 (20)	0.482
Diabetes mellitus, *n* (%)	24 (24)	9 (20)	15 (28)	0.359
Coronary disease, *n* (%)	20 (20)	5 (11)	15 (28)	0.044
Peripheral vascular disease, *n* (%)	17 (17)	6 (13)	11 (20)	0.426
Alcohol use, *n* (%)	20 (20)	8 (17)	12 (22)	0.621
Active smoking, *n* (%)	54 (54)	24 (52)	30 (55)	0.841
Immunosuppression, *n* (%)	10 (10)	6 (13)	4 (7)	0.506
Renal replacement therapy during ICU stay, *n* (%)	22 (22)	8 (17)	14 (26)	0.342
Vasoactive support during ICU stay, *n* (%)	53 (53)	23 (50)	30 (55)	0.422
Mechanical ventilation during ICU stay, *n* (%)	50 (50)	24 (52)	26 (48)	0.841
Limb amputation, *n* (%)	13 (13)	8 (17)	5 (9)	0.234
Mortality at day-28, *n* (%)	18 (18)	7 (15)	11 (20)	0.448
Mortality at day-90, *n* (%)	23 (23)	8 (17)	15 (28)	0.243

*BMI* body mass index, *HD* hemodynamic, *LRINEC* laboratory risk indicators for necrotizing fasciitis score, *SAPS-II* simplified acute physiology score-II, *SOFA* sequential organ failure assessment

**Table 2:** Relationship between the clinical and CT criteria and mortality at day-90

	Univariate analysis			Multivariate analysis			
	Alive at day-90 *n* = 39	Deceased at day-90 *n* = 15	*p* value	Odds ratio	5%	95% CI	*p* value
Age (years), median [IQR]	58 [51-67]	66 [56-68]	0.203				
Male sex, *n* (%)	26 (67)	11 (73)	0.751				
Obesity, *n* (%)	8 (21)	3 (20)	1				
Diabetes mellitus, *n* (%)	12 (31)	3 (20)	0.515				
Localization: limbs, *n* (%)	10 (26)	7 (47)	0.192				
Localization: pelvis, *n* (%)	10 (26)	6 (40)	0.333				
Localization: cephalic, *n* (%)	12 (31)	1 (7)	0.083				
Localization: trunk, *n* (%)	8 (21)	4 (27)	0.719				
Skin surface (%), median [IQR]	5 [5–9]	18 [9-19]	< 0.001	1.15	1.03	1.34	0.01
SOFA score on admission, median [IQR]	4 [1–7]	8 [3-10]	0.053				
SAPS-II on admission, median [IQR]	33 [21-52]	47 [40-56]	0.023				
LRINEC score on admission, median [IQR]	2 [1–5]	4 [3–6]	0.096				
Septic shock on admission, *n* (%)	23 (59)	12 (80)	0.208				
Delay of antibiotic initiation (days), median [IQR]	1 [0–4]	1 [0–3]	0.82				
CT characteristics							
Gas in the soft tissues	32 (82)	15 (100)	0.171				
Multiple fluid collections	6 (15)	8 (53)	0.012				
Absence or heterogeneity of tissue enhancement	33 (85)	15 (100)	0.17				
Significant inflammatory changes of the fascia and under	10 (26)	13 (87)	< 0.001	8.09	1.5	63.5	0.015

Multivariate analysis was performed with the selection of the best model according to the lower AIC by a stepwise logistic regression

*LRINEC* laboratory risk indicators for necrotizing fasciitis score, *SAPS-II* simplified acute physiology score-II, *SOFA* sequential organ failure assessment

In this study involving 100 severe ICU NSTI patients, we found that even if CT is not a diagnostic tool, it can nevertheless provide some information on the patient's outcome. It is well established that the contribution of CT for an NSTI diagnosis is not only unreliable but can also delay the management of the patient, which is why in most cases, the diagnosis is made on the basis of clinical examinations and operative findings [4]. Our work partly confirms these elements since, when comparing patients who had a CT-scan versus those who did not, no difference according to the initial severity was found.

There are many diagnostic studies in which changes in the fascia (thickness, presence of edema, nonenhancement) can help in the diagnosis, but to our knowledge, our study is the first to evidence a link between fascia imaging and prognosis [6]. Nevertheless, as the fascia is a key structure in the spread of infection, our findings seem quite consistent.

Our study has limitations, including its monocentric design with only 100 NSTI patients and a long cohort period. Of course, almost half of the patients in the cohort did not have a CT-scan, which is an undeniable source of bias. A multicenter prospective larger cohort study has to be performed to confirm these results.

## Data Availability

The datasets analyzed during the current study are not publicly available but are available from the corresponding author on reasonable request.

## Abbreviations

CT: Computed tomography
ICU: Intensive care unit
NSTI: Necrotizing soft-tissue infection
